# Predicting ipsilateral supraclavicular lymph node pathological complete response: nomogram based on the inflammatory markers

**DOI:** 10.3389/fonc.2024.1412607

**Published:** 2024-11-11

**Authors:** Chen Zhou, Xian Wu, Rongruo Lin, Li Xu, Tao He, Jinzhi Yi, Qing Lv

**Affiliations:** ^1^ Division of Breast Surgery, Department of General Surgery, West China Hospital, Sichuan University, Chengdu, China; ^2^ Breast Center, West China Hospital, Sichuan University, Chengdu, China; ^3^ Center of Infectious Diseases, West China Hospital of Sichuan University, Chengdu, China; ^4^ Department of Ultrasound, West China Hospital of Sichuan University, Chengdu, China

**Keywords:** inflammatory markers, ipsilateral supraclavicular lymph nodes (ISLNs), neoadjuvant chemotherapy (NAC), nomogram, pathological complete response (PCR), prognostic factors

## Abstract

**Background:**

The prediction of ISLN pCR after neoadjuvant chemotherapy (NAC) based on inflammatory markers and its prognostic value have rarely been investigated.

**Methods:**

Patients diagnosed with ISLN-involved breast cancer who received NAC in West China Hospital between September 2009 and December 2020 were enrolled in the derivation cohort for model construction and survival analysis, and patients with the same criteria between January 2021 and July 2024 were involved in validation cohort for external validation. After randomly dividing patients into training and testing groups at 7:3 ratio, a nomogram predicting ISLN pCR was constructed based on logistic regression in training group. Internal validation was performed in the testing group and external validation was performed in the independent validation cohort. The ROC curves were applied to validate the accuracy of the model. Survival analysis was performed using Kaplan−Meier plots.

**Results:**

A total of 120 eligible patients were involved in the derivation cohort to establish the nomogram (84 patients in training group and 36 patients in testing group), and 45 patients were involved in the independent validation cohort for external validation of the nomogram. Pretreatment NLR and hormone receptor (HR) status, as well as preoperative SII, CEA, CA15-3 and anti-HER2 therapy were included in the nomogram predicting ISLN pCR. The AUC were 0.906 (95% CI 0.837-0.975, P<0.001), 0.888 (95% CI 0.751-1.000, P<0.001) and 0.828 (95% CI 0.703-0.953, P< 0.001) in training, testing groups and the validation cohort respectively. ISLN pCR was significantly associated with better prognosis (all P<0.05).

**Conclusion:**

Inflammatory factors combined with tumor makers, hormone receptor status and anti-HER2 therapy could predict ISLN pCR effectively, which was significantly associated with improved survival outcomes.

## Introduction

According to Global Cancer Statistics 2020, breast cancer has exceeded lung cancer and become the most commonly diagnosed cancer worldwide (1). With advanced and emerging treatment therapies, the survival rate of breast cancer has improved. However, compared to the nearly 100% 5-year survival rate of stage I breast cancer, patients with ipsilateral supraclavicular lymph nodes (ISLN) involved (stage IIIc) often have unsatisfactory survival outcomes, whose 5-year survival rate decreases to 30-65.6% (2–5).

For ISLN metastasis, although it has been acknowledged to be local advanced breast cancer since the 6^th^ edition of the American Joint Committee on Cancer seventh edition (AJCC) TNM staging system defined ISLN metastasis as N3c, it still indicates poor prognosis (2–5). The critical role of systemic therapy—including chemotherapy, endocrine therapy, targeted therapy, and immunotherapy—has been well-established in the management of locally advanced breast cancer (LABC). Currently, neoadjuvant chemotherapy (NAC) has been more generally used in LABC patients or those with large breast cancer in order to decrease tumor burden and lessen node involvement, and NAC helps medication to distinguish chemoresistance simultaneously (6). Pathological complete response (pCR), defined through pathological and immunohistochemical (IHC) analysis following surgical procedure, is an important tool for prognostic prediction postoperatively. It is widely reported that pCR after NAC often indicates better survival prognosis in LABC patients (7), but the prognostic value of pCR in ISLN in patients with stage N3c disease is rarely discussed. Based on the limited reports, ISLN pCR was associated with a lower rate of relapse and a higher rate of disease-free survival when patients underwent ipsilateral supraclavicular lymph node dissection (ISLND), especially in triple-negative breast cancer (TNBC) (8, 9).

The evidence of the prognostic value of ISLN pCR is obvious, so distinguishing pCR in ISLN is of vital importance in LABC patients with ISLN metastasis. Although ISLN status after NAC could be accurately identified through surgical dissection and postoperative IHC analysis, it could result in more severe side effects postoperatively, lymphedema for instance (10). More importantly, most studies have shown that ipsilateral supraclavicular lymph node dissection (ISLND) does not result in better survival outcomes than radiotherapy (RT) (11–13). Nevertheless, supraclavicular radiation could also result in higher risk of lymphedema (10). Considering the treatment burden and long-term side effects of patients, the decision of treatment regimens should also be individual. For patients reached ISLN pCR after NAC, the treatment therapy for supraclavicular region could be considered to be simplified. Nevertheless, the way to predict ISLN pCR before local treatment for supraclavicular region (including ISLND and radiation) has rarely been investigated. Both Lv et al. (8) and Zhu et al. (9) found postoperative pathological information, including the number of axillary lymph node metastases, axillary pCR, breast pCR and Ki67 value could predict ISLN pCR, while these factors also could only be assessed after surgery. ISLN fine-needle aspiration (FNA) biopsy could help identify ISLN pCR, and the efficacy of identifying cancer-involved lymph nodes through FNA biopsy is unstable (14). As for assessment of ISLN status in non-invasive ways before surgery, scholars have investigated the predictive value of ultrasound, computed tomography (CT), and 18F-fluorodeoxyglucose (FDG) positron emission tomography/computed tomography (PET/CT) for predicting lymph node metastasis, indicating that PET/CT should be chosen for ISLN prediction, with a sensitivity of over 90% (15). However, PET/CT could burden patients with financial problems. As a result, an efficient model for predicting the status of ISLN after NAC before surgery is needed to help physicians make clinical decisions.

In recent years, inflammatory markers, including the neutrophil-to-lymphocyte ratio (NLR) and platelet-to-lymphocyte ratio (PLR), have been reported to have efficient value in predicting pCR after NAC in breast cancer patients (16–20). In patients with breast cancer, a low NLR or PLR was associated with a higher rate of breast cancer pCR after NAC (21, 22). The systemic immune-inflammation index (SII) is an inflammatory marker involving neutrophils, platelets and lymphocytes that is of vital importance in prognostic prediction in various malignant diseases (17, 23, 24), while the predictive value of SII on chemotherapy efficacy has rarely been reported. As for prediction of ISLN pCR after NAC, Liu et al. (25) investigated the prediction value of peripheral inflammatory index (PLT to lymphocyte ratio, PLR ratio) on ISLN pCR, but PLR had limited predictive value for pathological response of ISLN after NAC. Nevertheless, NAC could change the inflammatory status of patients. Both pretreatment and preoperative inflammatory indexes were reported to have prognostic value for both chemotherapy efficacy and survival outcomes in various malignant diseases, including breast cancer (26–28). As a result, the inflammatory status of different time point could also have prediction value of ISLN pCR, which was scarcely investigated.

Based on previous studies, the predictive value of inflammatory indexes for ISLN pCR has scarcely been discussed and there is still lack of a preoperative model for ISLN pCR prediction for LABC patients with ISLN metastasis after NAC. As a result, this study aimed to evaluate whether pretreatment and preoperative inflammatory indexes have predictive value for ISLN pCR and to further establish a predictive model for ISLN pCR after NAC. Additionally, the prognostic value of ISLN pCR was also investigated to explore the clinical importance of ISLN pCR prediction.

## Materials and methods

### Study population

This study included two retrospective and independent cohort, named derivation cohort and validation cohort. Respectively. Derivation cohort, used for model construction, internal validation and survival analysis, enrolled consecutive female patients diagnosed with primary breast cancer with ISLN metastasis in West China Hospital between September 2009 and December 2020 as study object. The diagnosis of breast cancer and ISLN metastasis was pathologically defined through preoperative biopsy or ISLND with radical surgery. The inclusion criteria were as follows: 1) patients with primary breast cancer; 2) pathologically defined ISLN metastasis; 3) patients who received NAC before breast surgery; and 4) patients who received ISLND. Patients were excluded from this analysis if they 1) had male breast cancer or 2) had distant metastasis, including metastasis to the bone, lung, or liver etc.; 3) bilateral breast cancer simultaneously or distant lymph node metastasis; 4) failure to be followed up for 6 months; and 5) lack of pretreatment or preoperative laboratory results.

As for validation cohort, used for external validation of the model. retrospectively reviewed the consecutive female patients diagnosed with primary breast cancer with ISLN metastasis in West China hospital from January 2021 to July 2024. The diagnosis of breast cancer and ISLN metastasis was in accordance with derivation cohort. The inclusion criteria of validation cohort included: 1) patients with primary breast cancer; 2) pathologically defined ISLN metastasis; 3) patients who received NAC before breast surgery; and 4) patients who received ISLND. Patients were excluded from validation cohort if they 1) had male breast cancer or 2) had distant metastasis, including metastasis to the bone, lung, or liver etc.; 3) bilateral breast cancer simultaneously or distant lymph node metastasis; 4) lack of pretreatment or preoperative laboratory results.

### Data collection and definition of inflammatory index

The baseline demographics, tumor burden, pretreatment pathological diagnosis, pretreatment and preoperative laboratory results, including inflammation biomarkers (neutrophils, lymphocytes, etc.), tumor markers [carcinoembryonic antigen (CEA) and carbohydrate antigen 15-3 (CA15-3)] and platelets, and treatments were retrospectively reviewed for every enrolled patient in derivation and validation cohort. All the involved individuals in derivation cohort received follow-up by telephone, outpatient visits, or rehospitalization every half year after hospital discharge.

Hormone receptors (HR), including estrogen receptor (ER) and progesterone receptor (PR), human epidermal growth factor receptor-2 (HER-2) and histological grade, were determined by IHC analysis through pretreatment aspiration. ER/PR positive were determined as 1% of tumor cells with nuclear staining with HR positive defined as ER or PR positive, and HER-2 positive was defined as 3+ by IHC staining or positive fluorescence *in situ* hybridization (FISH) results. Tumor grade was assessed in accordance with Elston and Ellis based on the combined assessment of tubule formation, nuclear grade, and mitotic activity.

Both the SII and NLR are inflammatory indexes based on neutrophils, platelets and lymphocytes, whose calculation formulas are neutrophils*platelet/lymphocytes and neutrophils/lymphocytes, respectively.

### Treatment therapy

All patients involved had received NAC before surgery. NAC regimens were divided into the following types: anthracycline-based, paclitaxel-based, anthracycline and paclitaxel combined and others. History of preoperative anti-HER2 therapy usage (including trastuzumab and pertuzumab) was also recorded. All the treatment strategies were based on both the assessment of experienced physicians and their own will. Axillary lymph node dissection (ALND) and ISLND were performed on every involved patient, and breast surgery, including mastectomy with/without contralateral prophylactic mastectomy and breast-conserving surgery (BCS), was performed according to the surgeons’ careful assessment and patients’ aspiration. Breast pCR was defined as a complete disappearance of all invasive tumor cells from breast tissue regardless of the presence of residual ductal carcinoma *in situ* (ypT0/is), and lymph node pCR was defined as no residual tumor cells in ALN or ISLN pathologically.

### Study outcomes

Breast, ALN and ISLN pCR was defined by pathological and IHC analysis after breast surgery and lymph node dissection. The survival outcomes were overall survival (OS), breast cancer-specific survival (BCSS) and disease-free survival (DFS). DFS was defined as the time from surgery to relapse, metastasis or death, BCSS was defined as the time from surgery to breast cancer-associated mortality, and OS was defined as the time from surgery to all-cause mortality.

### Statistical analysis

Eligible patients were randomly divided into training and testing groups (at a ratio of 7:3) using the “random” R package, and the basic characteristics were verified to be without significant differences (defined as P ≥ 0.05). The baseline demographics, tumor burden, pathological diagnosis, treatment strategies and laboratory results were compared between the two groups. Pearson’s chi-square test was used for categorical characteristics. For continuous variables, after the normal distribution test, the normally distributed data are expressed as the “mean ± standard deviation,” using Student’s t test for comparisons, while the abnormally distributed data are expressed as median values with interquartile ranges (IQRs) with a nonparametric test applied to carry out comparisons between the two groups. Continuous variables were standardized by standardized mean difference in the following analysis. Logistic regression was performed in the training group, and the factors with a P value <0.05 were involved in the construction of the nomogram predicting the possibility of ISLN pCR after NAC. Then, a nomogram was established to predict ISLN pCR in the training group and internal validation was performed in the testing group. For external validation, patients in validation cohort were involved merely for validate the effectiveness of the nomogram. The concordance index (C-index) and calibration curves were applied to measure the discrimination and accuracy of the model by the bootstrap validation method with 500 resamples in training, testing groups and the validation cohort. In addition, the receiver operating characteristic (ROC) curve was used to evaluate the predictive value of the nomogram in training and testing groups as well as the validation cohort, and the area under the ROC curve (AUC) was calculated.

Regarding survival outcomes, the time course for the occurrence of adverse outcomes after surgery in inpatients with ISLN metastasis was depicted as Kaplan–Meier curves to determine the prognostic value of ISLN pCR.

Statistical analyses were conducted by Statistical Package for the Social Sciences (SPSS) 25.0 and R software version 3.6.1 (http://www.rproject.org). A P value <0.05 was considered statistically significant.

## Results

### Patient characteristics

**Figure 1:**
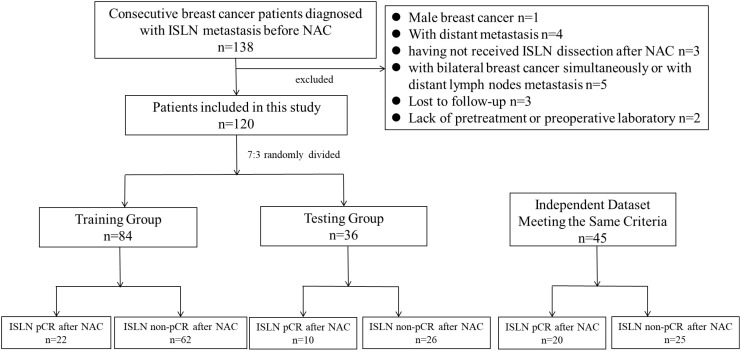
Flow chart of the study.

For derivation cohort, among the breast cancer patients administered in West China Hospital between September 2009 and December 2020, a total of 138 patients were pathologically diagnosed with ISLN metastasis before NAC, and patients were excluded from derivation cohort for the following reasons: (1) male breast cancer (n = 1); 2) distant metastasis (n = 4).; 3) having not received ISLND after NAC (n=3); 4) patients with bilateral breast cancer simultaneously or with distant lymph node metastasis (n=5); 5) failed to be followed up for 6 months (n=3); 6) patients without pretreatment or preoperative laboratory results (n=2). As a result, a total of 120 primary breast cancer patients with ISLN metastasis were included in the derivation cohort. Among the patients involved, 32 (26.7%) patients achieved ISLN pCR after NAC. Then, the patients involved were randomly divided into a training group (n=84) for model construction and a testing group (n=36) for internal validation, with ISLN pCR rates of 26.2% (22/84) and 27.8% (10/36), respectively (**Figure 1**). The median follow-up period was 40 months (5-142 months) in derivation cohort. After the same inclusion and exclusion of patients, a total of 45 patients hospitalized in West China hospital from January 2021 to July 2024 were involved in the validation cohort for external validation. The rate of ISLN pCR rates was 44.4% (20/45), which was significantly higher than the rate in derivation cohort (P= 0.029).

The characteristics of the included patients, including baseline information, comorbidities, tumor burden, treatment therapy and laboratory results, are described in **Table 1**. In the derivation cohort, the baseline characteristics of patients between training and testing groups were compared, it showed no significant difference between the two groups but merely CA15-3 before surgery showed significant variation (P=0.042). As for the validation cohort, the demography and characteristics of patients were compared to the derivation cohort, and the patients in the validation cohort had older age, more likely to receive contralateral mastectomy, longer NAC cycles and higher ALN and ISLN pCR rates (all P <0.05).

**Table 1:** Demographic and disease characteristics of patients.

Variables	Derivation Cohort	Training Group (n=84)	Testing Group (n=36)	P ^a^	Validation Cohort (n=45)	P ^b^
n	120	84	36		45	
**Age**	49.07 ± 8.19	48.83 ± 8.02	49.61 ± 8.69	0.636	52.51 ± 10.44	0.027
Immunological Comorbidities						
**Recent Blood Transfusion History (%)**				/		/
Yes	0 (0.0)	0 (0.0)	0 (0.0)		0 (0.0)	
No	120 (100.0)	72 (100.0)	36 (100.0)		45 (100.0)	
**Immunological Diseases (%)**				/		/
Yes	0 (0.0)	0 (0.0)	0 (0.0)		0 (0.0)	
No	120 (100.0)	72 (100.0)	36 (100.0)		45 (100.0)	
Tumor Burden						
**T (%)**				0.402		0.461
T0	2 (1.7)	1 (50.0)	1 (50.0)		0 (0.0)	
T1	13 (10.8)	8 (9.5)	5 (13.9).		4 (9.1)	
T2	57 (47.5)	37 (44.0)	20 (55.6)		21 (47.7)	
T3	23 (19.2)	17 (20.2)	6 (16.7)		5 (11.4)	
T4	25 (20.8)	21 (25.0)	4 (11.1)		14 (31.8)	
**N (%)**				/		
N3c	120 (100)	84 (100)	36 (100)		45 (100.0)	
Pretreatment Histological Characteristics						
**Histological Subtype (%)**				0.142		0.055
IDC	97 (80.8)	65 (77.4)	32 (88.9)		41 (93.2)	
Others	23 (19.2)	19 (22.6)	4 (11.1)		3 (6.8)	
**ER Status (%)**				0.686		0.748
positive	70 (58.3)	48 (57.1)	22 (61.1)		25 (55.6)	
negative	50 (41.7)	36 (42.9)	14 (38.9)		20 (44.4)	
**PR Status (%)**				0.354		0.392
positive	69 (57.5)	46 (54.8)	23 (63.9)		22 (50.0)	
negative	51 (42.5)	38 (45.2)	13 (36.1)		22 (50.0)	
**HR Status (%)**				0.228		0.450
positive	77 (64.2)	51 (60.7)	26 (72.2)		26 (57.8)	
negative	43 (35.8)	33 (39.3)	10 (27.8)		19 (42.2)	
**HER-2 Status (%)**				0.543		
positive	48 (40.0)	31 (36.9)	17 (47.2)		17 (37.8)	
negative	67 (55.8)	49 (58.3)	18 (50.0)		28 (62.2)	
uncertain	5 (4.2)	4 (4.8)	1 (2.8)		0 (0.0)	
**Grade**				0.886		0.008
II	33 (27.5)	22 (26.2)	11 (30.6)		24 (53.3)	
III/IV	49 (40.8)	35 (41.7)	14 (38.9)		12 (26.7)	
uncertain	38 (31.7)	27 (32.1)	11 (30.6)		9 (20.0)	
Treatment Therapy						
**NAC Regimen (%)**				0.035		0.018
Anthracycline-based	37 (40.8)	31 (36.9)	6 (16.7)		5 (11.1)	
Paclitaxel-based	18 (15.0)	11 (13.1)	7 (19.4)		8 (17.8)	
Anthracycline and Paclitaxel combined	53 (44.2)	37 (44.0)	16 (44.4)		21 (46.7)	
Others	12 (10.0)	5 (6.0)	7 (19.4)		11 (24.4)	
**NAC Cycles (%)**				0.282		<0.001
< 4 cycles	10 (8.3)	9 (10.7)	1 (2.8)		0 (0.0)	
4-6 cycles	87 (72.5)	58 (69.0)	29 (80.6)		25 (55.6)	
>6 cycles	23 (19.2)	17 (20.2)	6 (16.7)		20 (44.4)	
**Anti-HER2 Regimen (%)**				0.690		0.201
Yes	24 (20.0)	16 (19.0)	8 (22.2)		17 (37.8)	
No	96 (80.0)	68 (81.0)	28 (77.8)		28 (62.2)	
**Breast Surgery (%)**				0.203		0.014
BCS	2 (1.7)	2 (2.4)	0 (0)		1 (2.2)	
mastectomy	113 (94.2)	77 (91.7)	36 (100)		36 (80.0)	
contralateral mastectomy	5 (4.2)	5 (6.0)	0 (0)		8 (17.8)	
**Postoperative Radiotherapy (%)**				0.082		0.253
Yes	106 (88.3)	77 (91.7)	29 (80.6)		35 (81.4)	
No	14 (11.7)	7 (8.3)	7 (19.4)		8 (18.6)	
Laboratory Results						
Pre-treatment						
neutrophils	3.92 (2.14)	3.93 (2.37)	3.91 (1.85)	0.731	3.56 (1.92)	0.119
lymphocytes	1.65 (0.82)	1.66 (0.73)	1.63 (0.88)	0.49	1.68 (0.6)	0.727
platelet	193.5 (88.5)	193.5 (89.25)	195.0 (88.50)	0.817	220.0 (82.50)	0.157
SII	437.07 (389.04)	441.71 (354.35)	420.04 (430.34)	0.606	441.43 (228.08)	0.733
NLR	2.32 (1.3)	2.41 (1.09)	1.97 (1.44)	0.41	2.06 (1.09)	0.250
Pre-operative						
neutrophils	2.63 (2.25)	2.51 (2.35)	2.76 (1.82)	0.966	2.77 (1.63)	0.438
lymphocytes	1.23 (0.5)	1.21 (0.46)	1.37 (0.6)	0.168	1.22 (0.59)	0.632
platelet	197.5 (102.75)	190.5 (93.5)	211 (126)	0.254	188 (70)	0.663
SII	390.51 (385.08)	383.42 (379.6)	408.41 (397.37)	0.832	429.6 (346.57)	0.997
NLR	2.04 (2.3)	2.12 (2.48)	1.76 (1.82)	0.443	2.08 (1.68)	0.690
CEA<2.2	58 (48.3)	40 (47.6)	18 (50.0)	0.881	26 (57.8)	0.280
CA15-3<18	57 (47.5)	45 (53.6)	12 (33.3)	0.042	34 (75.6)	0.001
NAC Efficacy						
Breast pCR	18 (15.0)	14 (16.7)	4 (11.1)	0.435	8 (17.8)	0.663
Axillary pCR	15 (12.5)	9 (10.7)	6 (16.7)	0.366	14 (34.1)	0.002
ISLN pCR	32 (26.7)	22 (26.2)	10 (27.8)	0.857	20 (44.4)	0.029
Total pCR	7 (5.8)	4 (4.8)	3 (8.3)	0.444	5 (11.1)	0.245

IDC, invasive ductal carcinoma; ER, estrogen receptor; PR, progesterone receptor; HR, hormone receptor; HER-2, human epidermal growth factor receptor-2; BCS, breast conserving surgery; SII, systemic Immune-inflammation Index; NLR, neutrophil-to-lymphocyte ratio; CEA, carcinoembryonic antigen; CA15-3, carbohydrate antigen 15-3; pCR, pathological complete response.

Data are presented as the number of patients (%) and mean ± SD.

a P value refers to the comparison between training and testing groups.

b P value refers to the comparison between derivation and validation cohort.

P < 0.05 was considered statistically significant.

### Construction and validation of the nomogram predicting ISLN pCR

**Figure 2:**
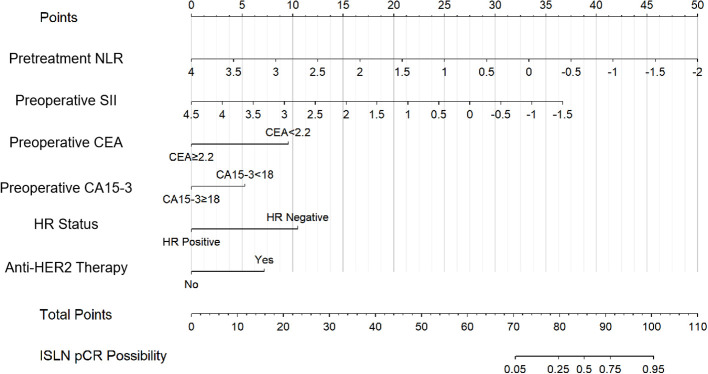
Nomogram for predicting ISLN pCR in patients with ISLN-involved breast cancer.

According to univariate logistic regression in the training group, patients with ISLN pCR after NAC had significant differences in the prevalence of lower pretreatment NLR (P=0.011), preoperative SII (P=0.033), CEA (cutoff set as 2.2, P=0.028) and CA15-3 (cutoff set as 18, P=0.013), pretreatment HR status (P=0.030) and anti-HER2 therapy usage (P<0.001, shown in **Table 2**). As a result, six variables (NLR, SII, CEA, CA15-3, HR status and anti-HER2 therapy usage) were incorporated into the nomogram. Based on the nomogram, points were signed for each variable, and a cumulative point could be calculated based on the patient’s pretreatment or preoperative inflammatory indexes (NLR and SII), tumor biomarkers (CEA and CA15-3), pathological results (HR status) and preoperative regimen (anti-HER2 therapy), in which lower scores were associated with a lower possibility of ISLN pCR (**Figure 2**). The nomogram showed that patients who had a lower pretreatment NLR, preoperative SII, CEA < 2.2, CA15-3 < 18, HR negative and having received preoperative anti-HER2 therapy were more likely to achieve ISLN pCR after NAC.

**Table 2:** Univariate logistic regression for ISLN-pCR in training group of breast cancer patients with ISLN metastasis.

Variables	ISLN-pCR Group	ISLN non-pCR Group	ISLN-pCR	
			*HR*, 95% CI	P^a^
**Age**			1.03 (0.97, 1.10)	0.312
Pretreatment Histological Characteristics				
Histological Subtype				
Others	6 (27.3)	13 (21.0)	Ref	
IDC	16 (72.7)	49 (75.4)	0.71 (0.23,2.17)	0.545
HR Status				0.030
HR negative	13 (59.1)	20 (32.3)	Ref	
HR positive	9 (40.9)	42 (67.7)	0.33 (0.12, 0.90)	
HER-2 Status				0.14
HER-2 negative	9 (40.9)	40 (64.5)	Ref	
HER-2 positive	12 (54.5)	19 (30.6)	2.81 (1.01, 7.80)	
HER-2 Uncertain	1 (4.5)	3 (4.8)	1.48 (0.14, 15.94)	
Grade				0.315
II	4 (18.2)	18 (29.0)	Ref	
III/IV	12 (54.5)	23 (37.1)	2.35 (0.65, 8.52)	
Hard to Determine	6 (27.3)	21 (33.9)	1.29 (0.31, 5.28)	
Preoperative Treatment				
NAC Cycle				0.434
< 4 cycles	1 (4.5)	8 (12.9)	0.36 (0.04, 3.11)	
4-6 cycles	15 (68.2)	43 (69.4)	Ref	
>6 cycles	6 (27.3)	11 (17.7)	1.56 (0.49, 4.97)	
Preoperative Anti-HER2 therapy				<0.001
No	11 (50.0)	57 (91.9)	Ref	
Yes	11 (50.0)	5 (8.1)	11.40 (3.30, 39.34)	
Laboratory Results				
Pretreatment				
neutrophils	3.44 (1.87)	4.15 (2.47)	0.449 (0.164, 1.226)	0.118
lymphocytes	1.83 (0.88)	1.59 (0.64)	1.294 (0.812, 2.06)	0.278
platelet	192.5 (86.5)	195 (90)	1.067 (0.664, 1.716)	0.788
SII	374.29 (186.54)	494.51 (411.83)	0.551 (0.285, 1.067)	0.077
NLR	1.89 (1.03)	2.67 (1.04)	0.364 (0.167, 0.793)	**0.011**
Preoperative				
neutrophils	2.1 (1.56)	2.66 (2.48)	0.437 (0.165, 1.155)	0.437
lymphocytes	1.19 (0.54)	1.22 (0.45)	1.249 (0.79, 1.975)	0.342
platelet	178 (95.75)	196.5 (97.5)	0.615 (0.342, 1.104)	0.104
SII	296.85 (126.65)	495.85 (614.25)	0.253 (0.071, 0.898)	**0.033**
NLR	1.61 (1.13)	2.49 (2.59)	0.371 (0.126, 1.091)	0.072
CEA<2.2	15 (68.2)	25 (40.3)	3.171 (1.131, 8.89)	**0.028**
CA15-3<18	17 (77.3)	28 (45.2)	4.129 (1.353, 12.597)	**0.013**

*HR*, hazard ratio; IDC, invasive ductal carcinoma; HR, hormone receptor; HER-2, human epidermal growth factor receptor-2; SII, systemic Immune-inflammation Index; NLR, neutrophil-to-lymphocyte ratio; CEA, carcinoembryonic antigen; CA15-3, carbohydrate antigen 15-3; pCR, pathological complete response.

a P < 0.05 was considered statistically significant. The bold values refers to P <0.05.

**Figure 3:**
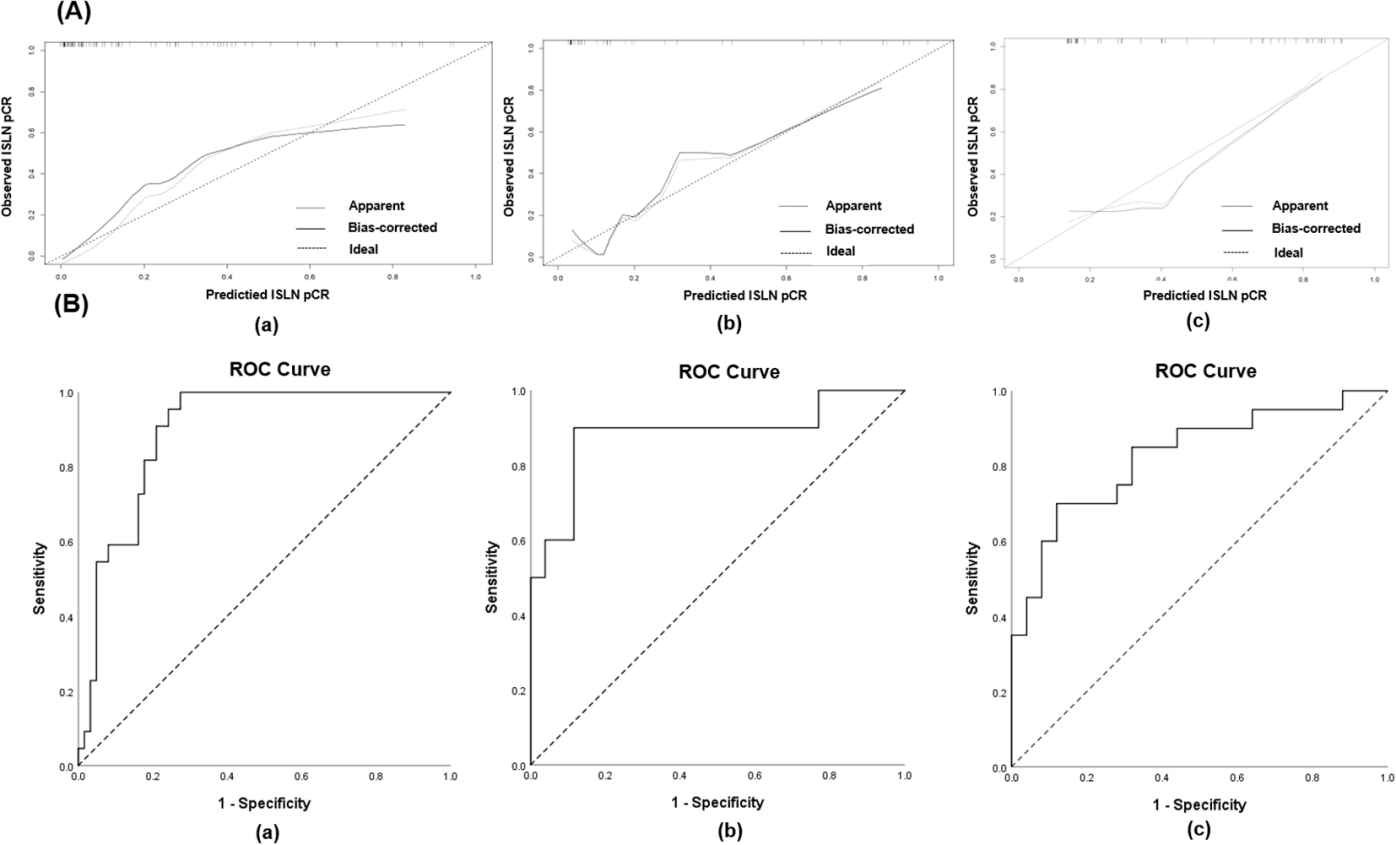
**(A)** Calibration curves for validating the nomogram in (a) training group, (b) testing group and (c) validation cohort; **(B)** The receiver operating curve (ROC) of the nomogram predicting ISLN pCR in (a) training group, (b) testing group and (c) validation cohort.

The nomogram was validated internally and externally using the testing group and validation cohort. The C-index of the nomogram in the training, testing groups and the validation cohort were 0.906 (95% CI: 0.838- 0.975), 0.888 (95% CI: 0.753-1.023) and 0.828 (95% CI: 0.705- 0.951) respectively, and the calibration curves for ISLN pCR predictions of the model are shown in **Figure 3A**. The receiver operating curve (ROC) of the nomogram predicting ISLN pCR in the training, testing groups and the validation cohort is depicted in **Figure 3B**. The areas under the ROC curve (AUCs) of the nomogram in the training, testing groups and the validation cohort were 0.906 (95% CI 0.837-0.975, P<0.001), 0.888 (95% CI 0.751-1.000, P<0.001), and 0.828 (95% CI 0.703-0.953, P< 0.001) respectively. Both the internal validation and the external validation showed a sufficient accuracy of the model.

### Survival analyses and prognostic factors of survival outcomes

**Figure 4:**
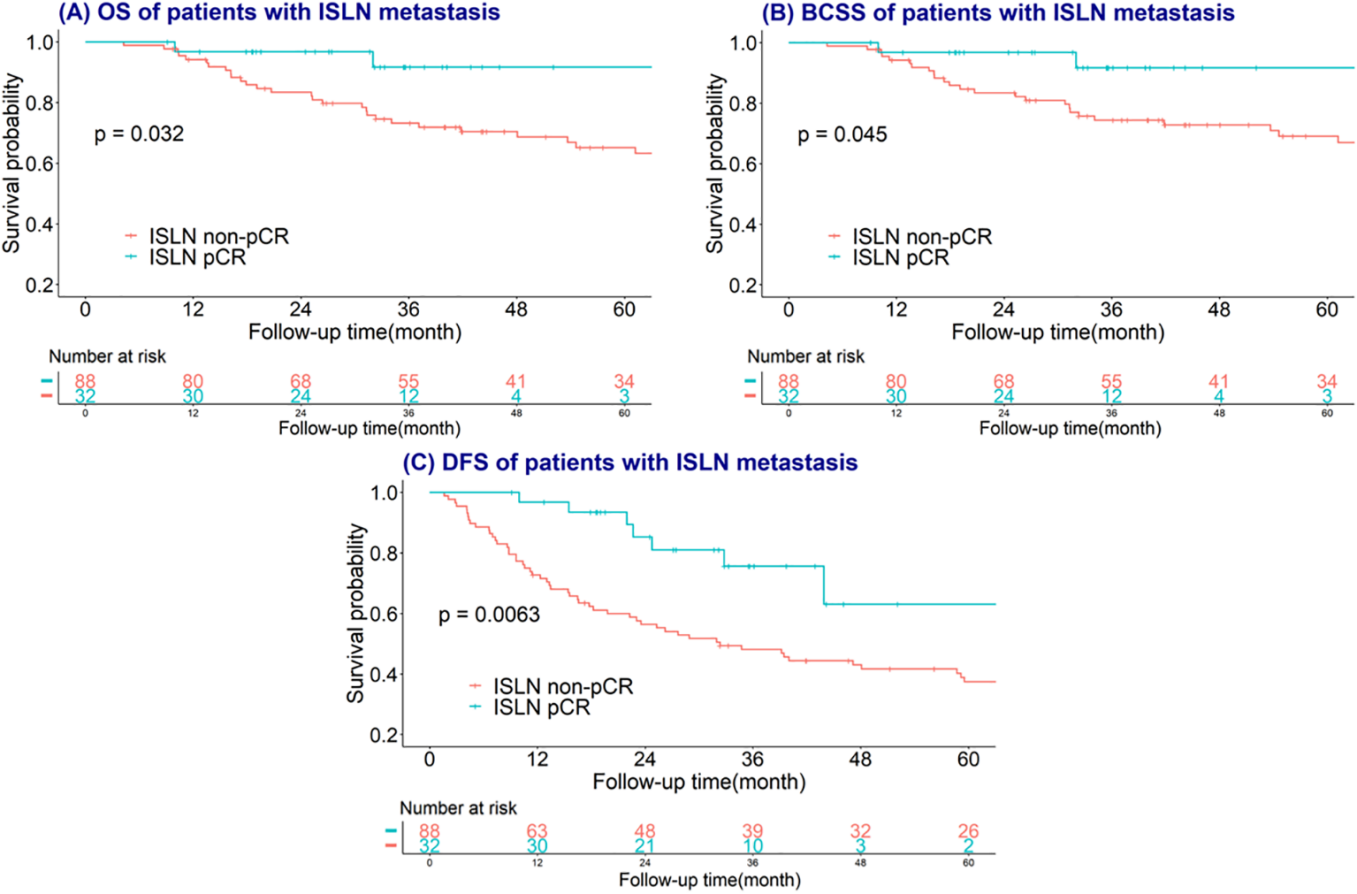
Survival curves plotted by the Kaplan–Meier method of ISLN pCR on **(A)** overall survival (OS); **(B)** breast cancer-specific survival (BCSS); **(C)** disease-free survival (DFS).

The median follow-up time of involved study population were 40 months (range from 5-148 months). Kaplan–Meier and log-rank tests were used to investigate the predictive value of breast, axillary, ISLN and total pCR on OS, BCSS and DFS, and the time-to-event analysis showed that ISLN pCR after NAC could predict OS, BCSS and DFS in ISLN-involved breast cancer patients. Patients with ISLN pCR after NAC showed significant better OS, BCSS and DFS (all P<0.05, shown in **Figure 4**). Nevertheless, breast, axillary, and total pCR could scarcely predict the prognosis outcomes in these patients (all P >0.05, shown in **Supplementary Figures 1**–**3**). To further evaluate the prognostic value of ISLN pCR after NAC, Cox analysis was applied to detect independent factors of adverse prognosis, including DFS. The variables with p values <0.05 according to univariate Cox analysis were included in multivariate Cox analysis (**Table 3**). Based on the multivariate Cox analysis, ISLN pCR was independently associated with longer DFS (ISLN pCR vs. ISLN non-pCR: *HR*=0.30; 95% CI, 0.11-0.81, P =0.018). Other than ISLN status after NAC, ER positivity, preoperative CEA<2.2 as well as postoperative intravenous chemotherapy were also independently associated with better DFS (ER positive vs. negative: *HR*=0.47; 95% CI, 0.27-0.81, P=0.006; CEA <2.2 vs. CEA≥2.2: HR= 0.50, 95% CI, 0.29-0.87 P =0.014; oral vs. intravenous chemotherapy: *HR* = 3.21, 95% CI, 1.58-6.50 P=0.01).

**Table 3:** Cox regression for disease-free survival in breast cancer patients with ISLN metastasis.

Variables	Univariate Cox Regression		Multivariate Cox Regression	
	*HR*, 95% CI	P^a^	*HR*, 95% CI	P^a^
**Age**	1.01 (0.98, 1.04)	0.547		
Chemotherapy Efficacy				
ISLN pCR	0.35 (0.16, 0.77)	**0.009**	**0.30 (0.11, 0.81)**	**0.018**
Breast pCR	0.4 (0.14, 1.1)	0.075		
Axillary pCR	0.46 (0.17, 1.26)	0.131		
Total pCR	0.24 (0.03, 1.72)	0.154		
Histological Characteristics				
Histological Subtype				
Others				
IDC	0.79 (0.42, 1.45)	0.44		
ER Status				
ER negative				
ER positive	0.58 (0.35, 0.97)	**0.039**	**0.47 (0.27, 0.81)**	**0.006**
PR Status				
PR negative				
PR positive	0.61 (0.37, 1.03)	0.065		
HER-2 Status		0.319		
HER-2 negative				
HER-2 positive	0.67 (0.39, 1.15)			
HER-2 Uncertain	1.1 (0.34, 3.55)			
Grade		0.424		
II				
III/IV	1.55 (0.81, 2.96)			
Hard to Determine	1.33 (0.67, 2.63)			
Laboratory Results				
Pretreatment				
neutrophils	0.96 (0.73, 1.28)	0.800		
lymphocytes	0.97 (0.74, 1.27)	0.799		
platelet	0.97 (0.74, 1.27)	0.823		
SII	0.92 (0.71, 1.19)	0.530		
NLR	0.95 (0.74, 1.23)	0.713		
Preoperative				
neutrophils	1.23 (0.97, 1.57)	0.092		
lymphocytes	0.84 (0.63, 1.13)	0.261		
platelet	0.93 (0.72, 1.19)	0.541		
SII	1.07 (0.84, 1.37)	0.581		
NLR	1.24 (0.98, 1.57)	0.076		
CA15-3<18	0.84 (0.5, 1.41)	0.506		
CEA<2.2	0.39 (0.23, 0.67)	**0.001**	**0.50 (0.29, 0.87)**	**0.014**
Treatment				
Breast Surgery		0.442		
BCS	Ref			
mastectomy	0.53 (0.13, 2.17)			
contralateral mastectomy	0.93 (0.16, 5.58)			
Postoperative Radiotherapy		0.695		
No	Ref			
Yes	0.85 (0.39, 1.88)			
Postoperative Chemotherapy		0.006		0.003
Intravenous	Ref		Ref	
Oral	2.62 (1.34, 5.15)		**3.21 (1.58, 6.50)**	
None	0.54 (0.19, 1.51)		0.86 (0.30, 2.51)	
Anti-HER2 Therapy		0.045		0.482
No	Ref		Ref	
Yes	0.50 (0.25, 0.98)		0.75 (0.34, 1.67)	

*HR*, hazard ratio; IDC, invasive ductal carcinoma; ER, estrogen receptor; PR, progesterone receptor; HER-2, human epidermal growth factor receptor-2; SII, systemic Immune-inflammation Index; NLR, neutrophil-to-lymphocyte ratio; CEA, carcinoembryonic antigen; CA15-3, carbohydrate antigen 15-3; pCR, pathological complete response; BCS, breast conserving surgery.

a P < 0.05 was considered statistically significant. The bold values refers to P <0.05.

## Discussion

This study developed a nomogram to predict ISLN pCR following NAC in primary breast cancer patients with pathologically confirmed ISLN metastasis, and evaluated the prognostic significance of ISLN pCR in this cohort. After randomly dividing the involved patients, the nomogram was constructed using the training group of derivation cohort and subsequently validated in both testing group of derivation cohort and validation cohort. Notably, ISLN pCR was significantly associated with improved overall survival (OS), breast cancer-specific survival (BCSS), and disease-free survival (DFS), underscoring its critical role in predicting outcomes.

In this study, a total of 120 patients, 26.7% (32/120) of whom achieved ISLN pCR after NAC, as well as 15.0% (18/120), 12.5% (15/120) and 5.8% (7/120) of whom achieved breast, axillary and total pCR, respectively. The pCR rate in this study was lower than that reported, in which a total pCR rate of 14.6-31.2% was observed (29–31). Compared with other studies regarding ISLN pCR, 54.4% ISLN pCR was observed in 307 patients with ISLND (8). The possible reason for the difference could be that the patients involved in this study were from 2009, and in the early period, the NAC regimen was relatively backward, which could result in lower pCR rates. On the other hand, those patients with suspected ISLN metastasis without pathologic confirmation, while after ISLND, no ISLN metastasis was observed, were excluded. In this way, possible ISLN pCR patients were excluded.

Consistent with prior reports, ISLN pCR was found to be more readily achievable than breast or axillary pCR. In terms of ISLN pCR prediction, Lv et al. (8) built a nomogram based on postoperative pathological information, including the number of axillary lymph node metastases and breast pCR, which were difficult to assess before surgery and also unrealistic to help make a decision on treatment therapies. Similarly, Zhu et al. (9) found that breast pCR, axillary pCR and Ki67 value could predict ISLN pCR, while these factors also could only be assessed after surgery. Although these studies noticed the necessity of prediction of ISLN pCR for these patients, the models could merely be used after the entire surgery and until the whole pathological results were issued. Other than nomogram regarding ISLN pCR, there were many nomograms built for breast cancer patients. In recent years, many nomograms combining radiomic information and clinical characteristics to predict pCR after NAC in breast cancer patients, which showed satisfactory prediction efficacy (32, 33). Further to predict survival, nomogram for predicting survival in patients with breast cancer were also common (34). These researches indicated that the prediction value of nomogram was applicable. Also, there’re some other predictive models related to therapy response in breast cancer patients (35, 36), but the prediction value of inflammatory factors on ISLN pCR after NAC was rare. Liu et al. (25) reported an unsatisfactory predictive function of the peripheral inflammatory index (PLT to lymphocyte ratio, PLR ratio) on ISLN pCR and did not construct a model for ISLN pCR prediction. In contrast, this study highlights the importance of pretreatment NLR and preoperative SII as key factors in predicting ISLN pCR after NAC. Based on logistic regression, a nomogram was built based on pretreatment NLR, HR status as well as preoperative SII, CEA, CA15-3 and receiving anti-HER2 therapy. As for other factors involved in the nomogram, CEA and CA15-3, known as vital tumor markers in breast cancer, could reflect tumor burden, and a high level of tumor markers could be associated with advanced disease and poor outcomes (37, 38). Also, it’s acknowledged that patients with negative HR status was more sensitive to NAC who were more likely to reach pCR (39). In patients with breast cancer expressing HER2, receiving anti-HER2 therapy could significantly raise pCR rates (40). The increasing use of adjuvant anti-HER2 therapy may explain the rise in pCR rates observed in our recent validation cohort.

The involvement of inflammatory factors in the nomogram predicting ISLN pCR after NAC was not baseless. According to previous studies, both low pretreatment and preoperative NLR were reported to have a significant association with a better pCR rate in breast cancer patients, especially in TNBC (18, 19). In recent years, SII has been more discussed as having value in predicting long-term survival outcomes in patients with malignant diseases (17, 23, 41, 42). Additionally, a low preoperative SII was found to have predictive value for chemotherapy efficacy across other cancer types (26–28). Nevertheless, the predictive value of SII for pCR after NAC has rarely been discussed. Chen et al. (43) explored the correlation of SII and NAC safety and efficacy in breast cancer patients, showing that there was no significant association between pretreatment SII and Miller and Payne grade (MPG). Additionally, a study on ISLN pCR post-NAC reported no significant association between inflammatory markers and ISLN pCR (25). SII integrates components of platelets, neutrophils, and lymphocytes, with NLR and PLR frequently cited as predictive markers for chemotherapy efficacy in patients undergoing NAC. In patients with breast cancer, low PLR was mostly reported to have predictive value of pCR after NAC (16, 17). Furthermore, Graziano et al. (21) further demonstrated that concurrent low PLR and NLR could predict favorable response to preoperative chemotherapy. Thus, SII may present a novel factor for pCR evaluation, as it partly combines platelets, neutrophils and lymphocytes. In fact, platelets are known to contribute to cancer progression, which could in some way represent the status of circulating tumor cells (CTCs) (44). An increase in circulating platelets may indicate tumor cell activation, as they can aggregate and interact with platelets to colonize secondary sites (45). Similarly, neutrophils have been reported to be associated with worse prognosis and unsatisfactory chemosensitivity (46). Additionally, the survival of neutrophils could be enhanced by breast cancer cell–neutrophil interactions and promote tumor metastasis through protumorigenic activities (47). Conversely, filtration of lymphocytes, serving an antitumor role surrounding tumor cells, usually predicts better prognosis (48), and a higher circulating lymphocyte count was associated with better prognosis in breast cancer patients (49). Furthermore, the post-treatment inflammatory index could also reflect the efficiency of chemotherapy as well as prognosis. According to Kim et al. (50), for patients using chemotherapy, patients with high NLR and PLR after treatment were associated with worse prognosis, indicating the vital prognostic value of the inflammatory index after NAC. Consequently, preoperative SII may offer valuable predictive insights into NAC effectiveness.

For patients with ISLN metastasis, consensus has not yet been reached regarding whether ISLND should be performed. As reported previously, dissection of the ISLN could scarcely improve prognosis compared to ISLN radiology (8, 11, 13, 51). Nevertheless, this study underscores an important prognostic value of ISLN pCR after NAC in this population, which correlates with improved OS, BCSS and DFS. Although there have been limited investigations on the prognostic value of ISLN pCR, this result was partly in accordance with a previous study, in which better DFS was observed (25). Nevertheless, merely ISLN pCR was observed to have prognostic value. The observed prognostic value of ISLN pCR may be partly influenced by the relatively small sample size. In our study, only 15.0% (18/120), 12.5% (15/120) and 5.8% (7/120) reached breast, axillary and total pCR respectively, which limits the dataset for total pCR analyses. To address this limitation, a prospective study involving breast cancer patients with ISLN metastasis was warranted. On the other hand, as previously reported, lymph nodes pCR after NAC was easier to reach and it might be a better prognostic indicator for survival outcomes in breast cancer patients (52). In addition, breast cancer related adverse outcomes were usually related to distant metastasis. As for ISLN metastasis, patients with ISLN metastasis exhibit similar prognosis to distant lymph nodes metastasis in breast cancer patients (3). Although ISLN metastasis is classified as local-regional disease, tumor cells in ISLN lesions may possess higher biological activity and greater metastatic potential. Achieving ISLN pCR may therefore suggest effective control of potential distant metastases. Consequently, assessing ISLN status post-NAC is critical. Although ISLND could help identify ISLN pCR after NAC, as an invasive procedure, surgery could have side effects. The nomogram presented in this study, which integrates peripheral inflammatory indices, tumor markers, HR status, and anti-HER2 therapy, aims to minimize patient harm and financial burden. In summary, assessment of ISLN pCR in a non-invasive way is of vital importance for breast cancer patients with ISLN metastasis, and this nomogram could be of great importance in clinical practice.

To our knowledge, this study is the first to discuss the predictive value of inflammatory markers on ISLN pCR and construct a nomogram predicting ISLN pCR combining both pretreatment and preoperative inflammatory markers. Inflammatory markers were easy to reach, with the advantages of being unharmful and easy to pay for. Moreover, this nomogram could help physicians assess patient prognosis after NAC preoperatively to make different individual treatment strategies. Nevertheless, this study had some limitations. First, this is a retrospective study, and selection bias could hardly be avoided. Most importantly, patients with suspicious ISLN metastasis but uncertain preoperative ISLN status were excluded, which could result in a low pCR rate in this population. Second, this was a single-center study, and the sample size was relatively low. Third, the nomogram was only validated in the validation cohort from the same center. External validation in another center is warranted to further validate the generalization of the prediction model.

According to the nomogram, combining pretreatment NLR, HR status and preoperative SII, CEA, CA15-3 and usage of anti-HER2 therapy could predict ISLN pCR in patients with ISLN metastasis receiving NAC. Additionally, we identified the prognostic value of ISLN pCR in this population. ISLN pCR after NAC was significantly associated with better OS, BCSS and DFS. Based on the results, it is vital to assess the chemotherapy efficacy of ISLN to make individual treatment decisions, and the predictive model is of great clinical significance. Further external validation and well-designed prospective trials with larger sample sizes are expected to provide more convincing evidence for ISLN pCR prediction in patients suffering from ISLN metastasis breast cancer.

## Conclusions

Inflammatory indexes, including pretreatment NLR and preoperative SII, had predictive value for ISLN pCR in breast cancer patients with ISLN metastasis before NAC. A nomogram involving pretreatment NLR, HR status and preoperative SII, CEA, CA15-3 and usage of anti-HER2 therapy could assist in predicting ISLN status preoperatively. ISLN pCR was significantly associated with improved OS, BCSS and DFS, and assessment of ISLN status after NAC was of vital clinical importance.

## Data Availability

The original contributions presented in the study are included in the article/**Supplementary Material**. Further inquiries can be directed to the corresponding author.

## Glossary

AJCC: American Joint Committee on Cancer seventh edition
ALN: axillary lymph node
ALND: axillary lymph node dissection
AUC: area under the ROC curve
BCS: breast-conserving surgery
BCSS: breast cancer-specific survival
CEA: carcinoembryonic antigen
CA15-3: Carbohydrate antigen 15-3
CT: computed tomography
C-index: concordance index
DFS: disease-free survival
ER: estrogen receptor
FDG: 18F-fluorodeoxyglucose
FISH: fluorescence *in situ* hybridization
FNA: fine-needle aspiration
HER-2: human epidermal growth factor receptor-2
HR: hormone receptor
*HR*: hazard ratio
IHC: immunohistochemical
ISLN: ipsilateral supraclavicular lymph nodes
ISLND: ipsilateral supraclavicular lymph node dissection
LABC: local advanced breast cancer
NAC: neoadjuvant chemotherapy
NLR: neutrophil-to-lymphocyte ratio
OS: overall survival
pCR: Pathological complete response
PET/CT: positron emission tomography/computed tomography
PLR: platelet-to-lymphocyte ratio
PR: progesterone receptor
RT: radiotherapy
ROC: receiver operating characteristic
SII: Systemic Immune-inflammation Index
SPSS: Statistical Package for the Social Sciences
TNBC: triple-negative breast cancer
95% CI: 95% confidence interval
