# Supplementary material for: Predicting ipsilateral supraclavicular lymph node pathological complete response: nomogram based on the inflammatory markers
Source: Front Oncol. 2024 Nov 11;14:1412607. doi: 10.3389/fonc.2024.1412607 (PMC11586358; doi:10.3389/fonc.2024.1412607)
Supplement: Supplementary file 1 [file DataSheet1.docx]

**
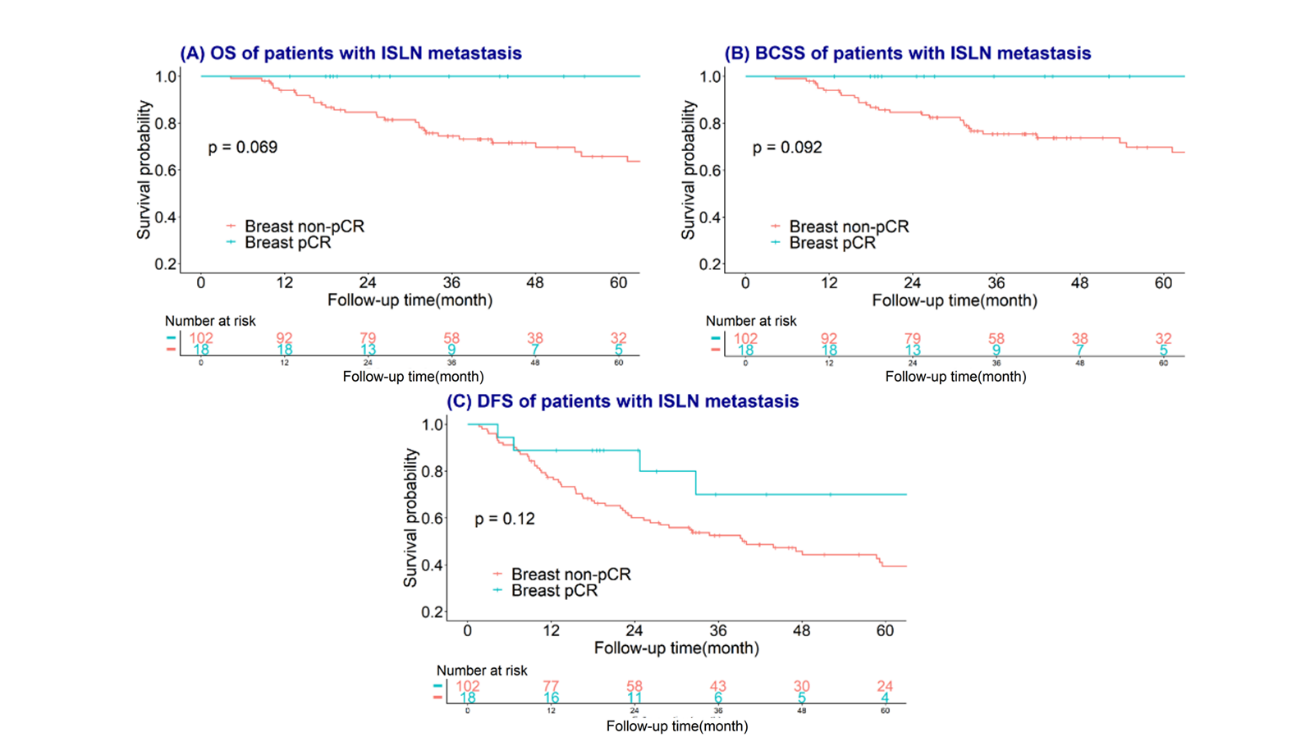
Supplemental Figure 1** Survival curves plotted by the Kaplan–Meier method of breast pCR on A) overall survival (OS); B) breast cancer-specific survival (BCSS); C) disease-free survival (DFS)

**
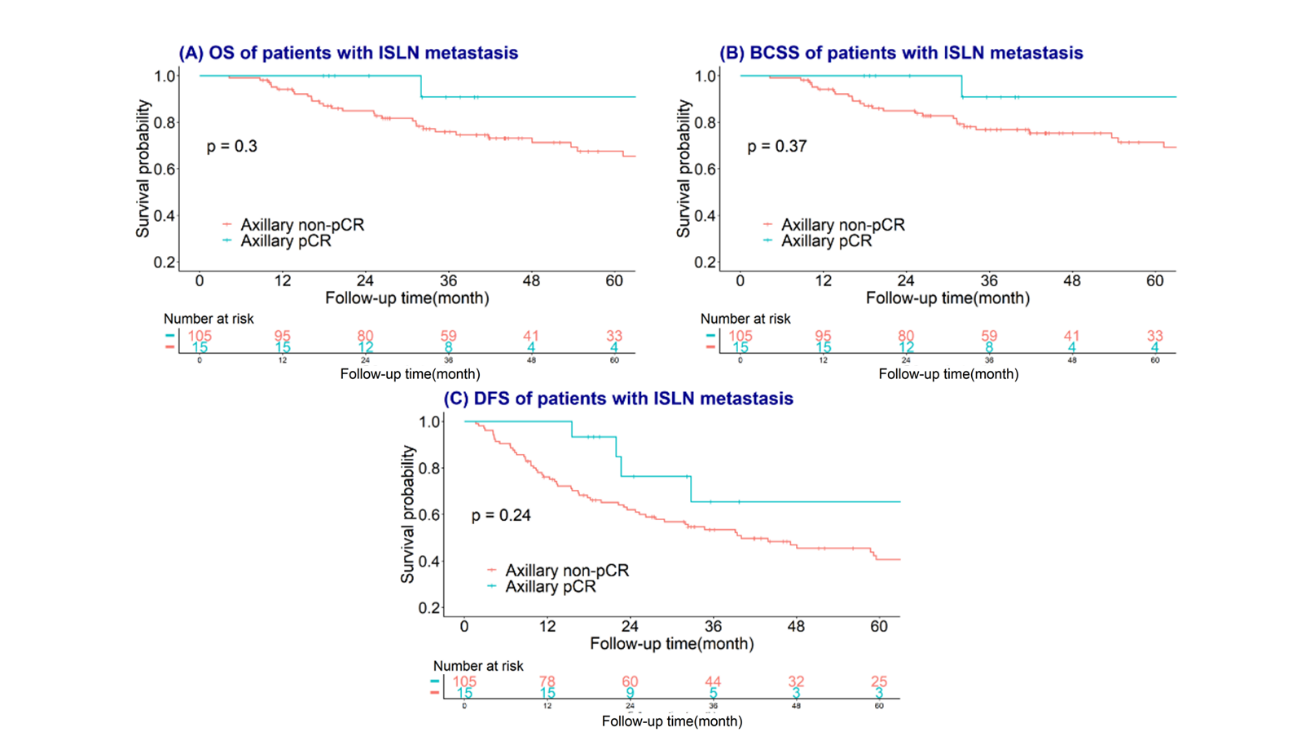
Supplemental Figure 2** Survival curves plotted by the Kaplan–Meier method of axillary pCR on A) overall survival (OS); B) breast cancer-specific survival (BCSS); C) disease-free survival (DFS)

**
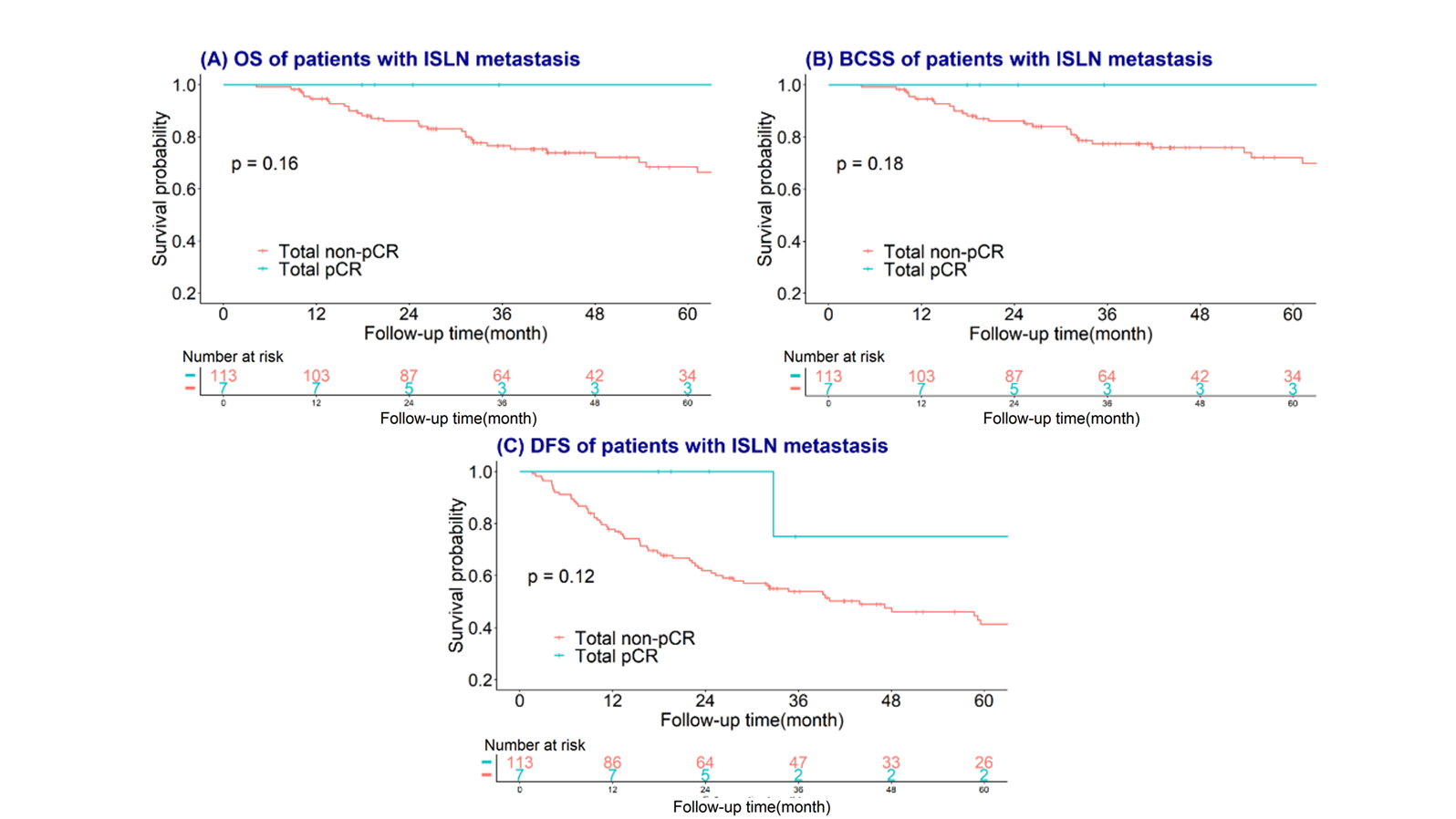
Supplemental Figure 3** Survival curves plotted by the Kaplan–Meier method of total pCR on A) overall survival (OS); B) breast cancer-specific survival (BCSS); C) disease-free survival (DFS)
